# The Dual Role of Calcium as Messenger and Stressor in Cell Damage, Death, and Survival

**DOI:** 10.1155/2010/546163

**Published:** 2010-03-15

**Authors:** Claudia Cerella, Marc Diederich, Lina Ghibelli

**Affiliations:** ^1^Laboratoire de Biologie Moléculaire et Cellulaire du Cancer, Hôpital Kirchberg, 9 rue Edward Steichen, Luxembourg L-2540, Luxembourg; ^2^Dipartimento di Biologia, Università di Roma Tor Vergata, Via Ricerca Scientifica snc, Rome 00133, Italy

## Abstract

Ca^2+^ is an important second messenger participating in many cellular activities; when physicochemical insults deregulate its delicate homeostasis, it acts as an intrinsic stressor, producing/increasing cell damage. Damage elicits both repair and death responses; intriguingly, in those responses Ca^2+^ also participates as second messenger. This delineates a dual role for Ca^2+^ in cell stress, making difficult to separate the different and multiple mechanisms required for Ca^2+^-mediated control of cell survival and apoptosis. Here we attempt to disentangle the two scenarios, examining on the one side, the events implicated in deregulated Ca^2+^ toxicity and the mechanisms through which this elicits reparative or death pathways; on the other, reviewing the role of Ca^2+^ as a messenger in the transduction of these same signaling events.

## 1. Introduction: Ca^2+^ Signaling versus Deregulation in Life and Death

Ca^2+^ is an ion involved in living processes in an atypical way: if other cations participate to enzyme activity without performing essential regulatory functions due to their abundance in all cell compartments, Ca^2+^ has a peculiar distribution, being present at very low levels in the cytosol of eukaryotic cells; this enables it to act as a messenger regulating cytosolic Ca^2+^-dependent enzymes and functions, when and where its local concentration raises above the steady-state level. For many decades the research on ion (and specifically Ca^2+^) roles in cell physiopathology was hampered by intrinsic technical difficulties. A big impulse came with the development of Ca^2+^-sensitive fluorescent probes [1, 2], that localize in specific cell compartments (cytosol, ER, and mitochondria) allowing separately and specifically evaluating and quantifying Ca^2+^ compartmentalization; and with the diffusion of instrumentations performing kinetic analyses, which allowed performing accurate and quantitative analysis of Ca^2+^ dynamics.

An efficient Ca^2+^ signaling implies maintenance of Ca^2+^  homeostasis, which requires mechanisms keeping cytosolic Ca^2+^ concentration ([Ca^2+^]c) low and stable. These include active pumping against gradient by Ca^2+^ ATPases, enzymes present on the cytosolic side of plasma membrane and endoplasmic reticulum (ER) performing the high energy-expensive task of pumping Ca^2+^ out of the cytosol against gradient, or by ion exchangers (e.g., the Na^+^/Ca^2+^) [3]. Instead, signaling is exerted by discrete and highly controlled ER membrane channels, originating local Ca^2+^ rises possessing specific signaling roles, such as those regulated by the phospholipase C-inositol-3-phosphate pathway [3], or by cyclic (ADP-ribose) [4]. These local increases activate/deactivate Ca^2+^-sensitive enzymes, eliciting signal transduction chains aimed at controlling many diverse cell functions such as mitosis, or activation, or motility or apoptosis. As a feedback mechanism, the channels rapidly close due to local high [Ca^2+^], which is rapidly extinguished by cytosolic buffering proteins, by the Ca^2+^-ATPases and by mitochondria, thus being in fact Ca^2+^ transients. To restore ER homeostasis, the partially emptied ER is replenished by Ca^2+^ entry from the extracellular space through controlled opening of plasma membrane channels (capacitative Ca^2+^ entry, see below [5]), or by Ca^2+^ released from mitochondria present in the vicinity of inositol-3-phosphate (IP_3_) channels [6]. In the category of excitable cells, mainly neurons and myocytes, plasma membrane channels open following specific hormonal or physico-chemical stimulations, even in the absence of previous ER emptying, and possess signaling meaning of their own [7].

Ca^2+^ signaling requires the strict cooperation among the different cellular compartments and organelles, being in fact a highly sophisticated way of communication to maintain homeostasis and functionality of the whole cell. In particular, much attention is being given to the cooperation between ER and mitochondria, which interact through highly dynamic physical connections [8] containing abundant Ca^2+^-mediating transport systems [9]. These mediate the controlled reciprocal exchange of Ca^2+^ [10] aimed at modulating and supporting each other functions in the guise of an interorganellar symbiotic relationship [11–13]. 

The important implication of the low cytosolic Ca^2+^ concentration ([Ca^2+^]c) for cell homeostasis is that it must be maintained low against gradient (extracellular space and the internal Ca^2+^ stores such as ER, have 10,000 times as much Ca^2+^), since excess or deregulated [Ca^2+^]c pose cells in such a dramatic asset to be in fact cell-toxic. To this purpose, a wide range of mechanisms are displayed, including high capacity binding proteins, pumps and exchangers, and mitochondria, which possess a low affinity Ca^2+^ uniporter that sequesters cytosolic Ca^2+^ when it reaches the dangerous threshold of 500 nM, thus being a major detoxifying mechanism against Ca^2+^ overload [14].

Failure of the mechanisms devoted to maintain Ca^2+^ homeostasis produces generalized Ca^2+^ alterations, in turn producing rough cell damage, without involving specific signaling meaning; when strong, Ca^2+^ alterations cause cell death by necrosis [15–19].

What becomes clear after many years of intense studies about the role of Ca^2+^ in cell stress response and death, is that pathological Ca^2+^ alterations resulting from failure of homeostatic controls coexist with regulated Ca^2+^ signaling. The former produces rough damage that leads to cell repair, or apoptosis, or necrotic cell death, according to the intensity of the damage, whereas the latter constitutes a controlled cell response participating in survival or apoptotic pathway. This recommends careful analyses to separate active responses from passive changes.

## 2. Ca^2+^ and Cell Damage

### 2.1. Ca^2+^ as an Intrinsic Stressor

Ca^2+^ deregulation is a consequence of many different insults that end up altering Ca^2+^homeostasis, causing and increasing damage to cells; for this reason, it may be defined as an “intrinsic stress,” meaning that it is autoinduced by the cells as a consequence of an extrinsic stress of a different nature. The proteins controlling Ca^2+^ homeostasis are so many and so diverse that it is quite likely that any insult or physico-chemical alterations end up deregulating some of them, producing a set of reactions that may not properly be defined as signaling (since it has no physiological purposes), but it is nonetheless obliged by the presence of Ca^2+^-sensitive determinants. What is lacking in such instances is the coordination between the multiple pathways, which are instead casually activated, overlapping and superimposing one another, leading to cell collapse. The intrinsic Ca^2+^ stress may consist in either depletion of ER Ca^2+^, or increase of cytosolic (or mitochondrial, or nuclear) Ca^2+^, or both.

### 2.2. Damage by Ca^2+^ Overload

When stress leads to Ca^2+^ overload, Ca^2+^-produced damage may reach levels sufficient to cause necrotic cell death [15–19]. Damage and death are due to excess stimulation of Ca^2+^-sensitive targets, which are numerous and concern key cellular functions: many enzymes that control supramolecular assembly, or degrading nucleic acids, lipids or proteins are Ca^2+^-sensitive. Among them are m-calpains, activated by high Ca^2+^ levels and implicated in cell death and in many neurological disturbances [20]; lipoxygenases, and a set of Ca^2+^-activated enzymes modifying arachidonic acid (AA) that are major actors of the inflammatory response, and also involved in apoptotic pathways [21]; phospholipases A2, which liberate AA from phospholipids, thus favoring, in the presence of high [Ca^2+^]mt mitochondria stress or collapse [22]; a set of DNAses, one of which historical interest for being the enzyme responsible for apoptotic laddering [23].

A major form of damage is caused by the intervention of mitochondria that, taking up the excess of cytosolic Ca^2+^ for scavenging purposes, may be subjected to stress and even collapse if it exceeds a physiological threshold, therefore increasing cell damage (see below) [14].

Another form of damage comes from energy failure, which starves Ca^2+^-ATPases that stop pumping Ca^2+^ against gradient from cytosol to ER, or to the extra-cellular environment, thus simultaneously producing cytosolic Ca^2+^ overload and ER Ca^2+^ depletion. 

Cytosolic Ca^2+^ overload is implicated in many serious human pathologies. 

Excitotoxicity is a major cause of neuronal cell death; it develops as a consequence of problems occurring during neurotransmission, in instances of excess of excitatory signals, such as those from the neurotransmitter glutamate [24], or of deregulated signaling; this ends up impairing the tight control of Ca^2+^ channels, leading to Ca^2+^ overload [25] and eventually cell death and neurodegeneration.

Ischemic and anoxic stress produce deep changes in cell metabolism that, upon reoxygenation/reperfusion converge into a dramatic, toxic increase of cytosolic Ca^2+^ [26, 27]. Such changes include plasma membrane depolarization, which favors the opening of the plasma membrane Ca^2+^ channels thus promoting Ca^2+^ influx [28]; and acidification, which causes the inversion of the Na^+^/Ca^2+^ plasma membrane exchanger, which begins pumping Ca^2+^ within cells [29]. Mitochondria may buffer Ca^2+^ and rescue cells in instances of mild reperfusion stress; however, they paradoxically are the major cause of cell death in strong reperfusion stress [30], since the huge Ca^2+^ overload stimulate Ca^2+^ overcharging and collapse through phenomena of Ca^2+^ cycling (see below [31]).

Due to the increasing evidence that most (if not all) pathologies involve, as etiological or concurrent agents, alterations of oxidative metabolism leading to oxidative stress, much attention was paid in the 80s and 90s to the mechanisms through which oxidative stress causes Ca^2+^ derangements. Although no definite picture is still delineated, some key points have been clarified. Oxidation and redox imbalance cause ER and plasma membrane Ca^2+^ channels malfunctions, since their oligomeric active form is controlled by disulfide bridges [32]; this increases [Ca^2+^]c and depletes [Ca^2+^]er. Moreover, oxidative stress impairs the buffering capacity of mitochondria, lowering the internal Ca^2+^ threshold level of PTP opening [9, 33], thus depriving the cells of one of the major Ca^2+^ detoxifying mechanisms. 

All of these derangements are especially critical for neurons, where Ca^2+^ is crucial to neuronal functions [34], implying that they possess more controlling steps that can be altered. Moreover, cell death is most devastating for tissues rich in post-mitotic cells, such as cardiomyocytes or neurons, which are difficult to replace; indeed, most neurodegenerative conditions are characterized by neuronal death caused by Ca^2+^ overload [34]. The scenario is even more dramatic considering that the organs that mostly depend on post-mitotic, Ca^2+^-sensitive cells are heart and brain, whose failure causes immediate death of the organism.

### 2.3. Ca^2+^ Overload in Mitochondria

Mitochondria are very important for intracellular Ca^2+^ homeostasis and signaling, acting in fact as pivot of intracellular Ca^2+^ communications. Any Ca^2+^ overload exceeding the cytosolic threshold of 500 nM involve mitochondrial participation [14]. Mitochondria possess low affinity (500 nM) Ca^2+^ uniporters that allow the accumulation of large amount of Ca^2+^ within the mitochondrial matrix, which constitutes a high capacity Ca^2+^ reservoir, allowing buffering [Ca^2+^]c increases over 500 nM [9, 14]. This mitochondria ability plays an important role in cell homeostasis and cell signaling, because it help extinguishing cytosolic Ca^2+^ signals [35]. The resulting [Ca^2+^]mt increase modulates mitochondrial activity (i.e., increases ATP production [9, 11, 12]); moreover, overcharged mitochondria helps refilling ER after physiological Ca^2+^ emptying (e.g., after IP_3_-mediated signalling [13]). In instances of mild [Ca^2+^]c increases originating from stressing events, potentially toxic Ca^2+^ is sequestered within mitochondria, and then released after the stress is over: in this instance mitochondria play a prosurvival role. However, if the amount of sequestered Ca^2+^ exceeds mitochondrial capacity, it leads to collapse through opening of the permeability transition pores (PTP, formerly referred to as megachannel) [18, 19]. Since PTP is a multi-ion channel, the consequence is that the captured Ca^2+^ ions are dissipated, creating new cytosolic Ca^2+^ increase, which can be in turn taken up by new intact mitochondria [18]. This creates cycles of Ca^2+^ uptake and dissipation, recruiting more mitochondria, up to a sort of mitochondrial suicide cascade. This phenomenon was named Ca^2+^ cycling [18], and raised much interest in the 80s; the interest then declined because it did not support a clear physiological role, being rather considered a futile cycle, because it does not help cells to survive. Nowadays, a re-evaluation of this mechanism suggests that Ca^2+^ cycling provides a physiological advantage [10]: PTP opening by itself causes release of cytochrome *c* (even in the absence of an upstream canonical apoptotic signaling such as Bax translocation [19, 36, 37]), which in turn may activate caspases and promote apoptosis [18], thus transforming a necrotic cell death into a more physio-compatible apoptosis. It seems worth mentioning here that localized phenomena of mitochondria Ca^2+^ cycling may have a pro-apoptotic signaling meaning since local and controlled small episodes of cytochrome *c* release act as initiators of the intrinsic pathway of apoptosis [38] (see below).

Mitochondria can adjust their cellular localization by moving around the microtubular network [39]; it is tempting to hypothesize that they reach positions required to perform Ca^2+^ detoxification, or to modulate specific signaling events, that is, extinguish some and exacerbating others, according to need. As an example, acute oxidative stress induces the reorganization of mitochondrial pattern from pan-cytoplasmatic, to peri-nuclear (Ghibelli, unpublished observation), possibly buffering excess ER Ca^2+^ leakage due to oxidations. This scenario suggests that local Ca^2+^ increases of a stress nature, even of a small extent, may trigger an apoptotic signaling *via* recruitment of mitochondria.

### 2.4. Damage by Ca^2+^ Depletion (ER Stress)

When referring to intracellular Ca^2+^ depletion, the emphasis goes to emptying of ER, which elicits what was recently recognized as ER stress [40]. ER stress is caused by different disturbances affecting ER homeostasis, such as protein malfolding, glucose starvation, disturbance of membrane turnover/synthesis, or of protein trafficking, which all lead to ER vesiculation and loss of function [40]. Ca^2+^ plays a key role in maintaining ER structure, since the flat shape of the cisternae is actively kept by bridges constituted by high capacity Ca^2+^ binding proteins such as calreticulin, calsequestrin, and calnexin [41]; upon ER Ca^2+^ emptying, Ca^2+^ binding is lost, the bridges weaken and ER resumes the low energy spherical shape of lipids droplets in acqueous solution, thus losing function. ER stress, as any other stress, can evolve into repair or apoptosis.

In the first instance, the stress response implies up-regulation of stress proteins such as GRP78, a major luminal ER protein [42] that plays a central role as ER stress sensor displaying multiple functions. It coordinates the activation of other proteins implicated in ER stress, such as ATF6 [43], a transcription factor transactivating prosurvival genes whose promoters containing ER stress response elements (ERSEs) [44]. GRP78 also promotes removal by autophagy of the altered portions of ER by controlling the correct formation of autophagosome [45]. Multifaceted is its ability to prevent apoptosis [42, 46]: a fraction of GRP78 is present as a transmembrane ER protein, exposing a cytoplasmic domain able to directly interact and form an inhibitory complex with caspase-7 and/or caspase-12 [42, 47]; GRP78 limits the pro-apoptotic activation of c-Jun N-terminal kinase (JNK) [35], normally acting as a transducer of ER stress [48]; in addition, cell-free studies suggest a direct ability of GRP78 to control mitochondria, by inhibiting cytochrome *c* release [47]. 

If damage is severe, it triggers apoptosis. The mechanism for ER stress-induced apoptosis is still not completely clarified. Big emphasis was given to caspase-12, which is activated in ER membranes in instances of disruption of ER Ca^2+^ homeostasis or accumulation of unfolded proteins in the ER lumen of mice cell models. Caspase-12 initiates apoptosis either in a mitochondrial-independent fashion [49] or recruiting and activating mediators of the intrinsic pathway of apoptosis [50]. This led to consider caspase-12 as the general transducer of ER damage. However, caspase 12 is present only in rodents, and to-date no functional caspase-12 was identified in human cells (which in fact possess only a pseudo-gene), nor a functionally equivalent protein. While the search for a human equivalent of caspase 12 is still active (especially concerning a possible role for caspase-4 [51]), other caspase-independent scenarios, have been explored to describe the transduction of the ER stress to apoptosis via mitochondria. Recently, Klee et al. [52] provided evidence that Ca^2+^ mobilization from the ER is required to initiate the mitochondrial death pathway, by cooperating with the effectors of ER stress surveillance machinery IREa/TRAF2; according to this model, Ca^2+^ promotes the JNK pro-apoptotic pathway through a complex set of steps involving the Bcl-2 family.

## 3. Ca^2+^ and Cell Death

### 3.1. Historical Perspective

When the regulated, physiological mode of cell death, apoptosis, came into the general interest, the involvement of de-regulated Ca^2+^ rises as causative agent of apoptosis was sought for, in the view that apoptosis was a sort of “petit necrosis;” in particular, a Ca^2+^/Mg^2+^-dependent DNAse was hypothesized, considering that DNA laddering was the earliest apoptotic marker of a biochemical nature to be widely accepted. The issue however never came to a definite picture, because if an increase of [Ca^2+^]c was indeed occurring in some examples of apoptosis, such as the paradigmatic model of rat thymocytes treated with corticosteroids [53], in many other instances it was a drop in overall Ca^2+^ level the event that promotes apoptosis [54]. 

The paradox of this dual, opposite role of Ca^2+^ in apoptosis occupied several years of research. Thapsigargin (THG), a (still) popular inducer of apoptosis, is an irreversible poison of ER Ca^2+^-ATPases (SERCA), thereby inducing a transient increase in cytosolic Ca^2+^ and a sustained depletion of the ER Ca^2+^ pool [55]; since the two events occur simultaneously, it is quite difficult to assess the specific contribution of one or the other to apoptosis. If the early studies were taking for granted that the apoptogenic event was the increase of [Ca^2+^]c, it was later shown that in many instances intracellular Ca^2+^ buffering did not abrogate, but even increased, THG-induced apoptosis, demonstrating that also ER Ca^2+^ depletion was an apoptogenic event [56, 57].

In fact, in most instances [Ca^2+^]c rises in apoptosis are not rough event, but precise signaling steps, such as, for example, the Ca^2+^-dependent proteases calpains [58] or calmodulin [59]. The notion of apoptotic Ca^2+^ signaling evolved, with not small effort, together with the awareness that apoptosis is not a “petit necrosis” but a regular signal transduction chain, occurring in functioning cells, ending up with coordinate cell demise instead of activation, or mitosis, or transcription. 

To-date, the issue of the actual role of Ca^2+^ in cell death is still debated; the different contributions of the different cells (e.g., excitable versus non-excitable; dividing versus post-mitotic; tumor versus normal) and of the different apoptogenic treatments (stress or physiologic; allowing or not protein neosynthesis, etc.) produce a whole continuum of variations, and render it hard to interpret the thousands of studies on the topic. However, some generalizations can be attempted: ER Ca^2+^ depletion may elicit apoptosis through the ER stress pathway [57, 60]; rough, stress-induced Ca^2+^ overloads produce necrosis [17, 19, 61]; regulated Ca^2+^ increases play a role as signaling events in the intrinsic apoptotic pathway [62–65].

The poor knowledge of the role of Ca^2+^ in apoptosis, which is perhaps surprising considering that Ca^2+^ dynamics were among the first alterations proposed as causative of apoptosis, is also due to other, more intrinsic problems. Among them the fact that Ca^2+^ transients are very much localized in terms of space (i.e., cytosolic micro-domains) and time (seconds), and it is very easy to miss them even with sophisticated technologies. Also the asynchrony of the apoptotic process hampers the analyses: even homogeneous cultured cells initiate apoptosis at different moments after stimulation, overlapping different phases and rendering inappropriate any biochemical analysis performed in bulk. These problems were overcome with technological approaches allowing analysis at the single cell level [1, 2], that is, living cell imaging and flow cytometry, which are beginning to shed light on the process, helping to separate different phases and different subregions of Ca^2+^ signaling [1, 2, 64, 65].

### 3.2. Stress-Induced Apoptosis: A General View

It is now well established that the intracellular apoptotic signaling evolves through at least two different pathways, triggered by ligand stimulation of death receptor (extrinsic pathway), or by cell damage (intrinsic pathway) [66]. The extrinsic pathway is a typical signal transduction consisting of protein-protein interaction and conformational changes from the very beginning, being induced by a molecular event such as ligand-receptor interaction and culminating with caspase activation and cell dismantling. The intrinsic pathway is instead induced not by molecular, but by physicochemical events, implying that (a) sensor(s) of micro-environmental alterations or cell damage must be activated to promote the apoptotic signal [67]; afterwards, a molecular signal transduction chain of events similar to the extrinsic pathway is activated, also culminating with caspase activation. Sensors are proteins that are modified by physico-chemical alterations such as pH, redox equilibrium, or Ca^2+^ levels, thus acquiring the ability to trigger a molecular signal cascade. The most upstream molecular event of the intrinsic pathway is the translocation of Bax, which moves to mitochondria and induces mitochondrial outer membrane permeabilization (MOMP). The difficulty of finding molecular events upstream of Bax activation suggested that Bax itself might be a sensor of physico-chemical alterations. Indeed, recent reports indicate that Bax activation can occur via direct oxidation of cysteines [68, 69], or via proteolytical activation by calpains [70].

### 3.3. Ca^2+^ Control of Cytochrome c Release

**Figure 1:**
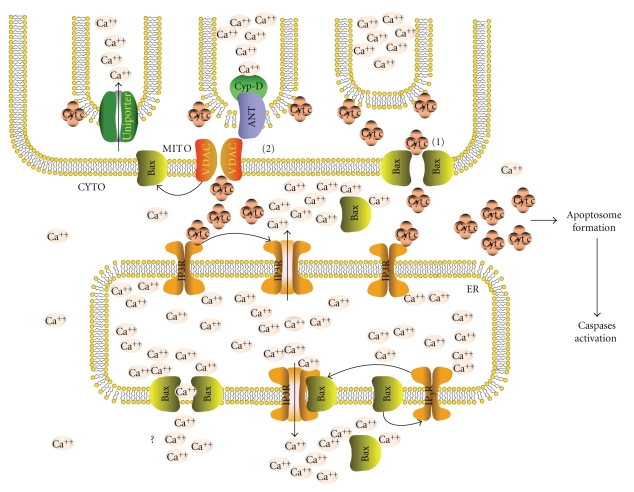
*Ca^2+^ signals between ER and mitochondria coordinate the precommitment phase of apoptosis:* amplification loops between Bax activation and Ca^2+^ release from ER amplify cytochrome *c* release to a level sufficient for apoptosome nucleation and caspase activation. Cytochrome *c* released by Bax (1) or VDAC (2) mitochondrial pores magnifies Ca^2+^ efflux from IP_3_ channels; the consequent local high cytosolic Ca^2+^ recruits Bax (*via* calpain?) to mitochondria or ER membrane, stimulating more cytochrome *c* release and more Ca^2+^ efflux, respectively.

It is emerging a pre- or early-commitment phase of the intrinsic apoptotic pathway, occurring before MOMP, during which potential apoptotic signals, mostly relying on Ca^2+^ messages, are selected and amplified by cross-talk between ER and mitochondria [61] (Figure 1). MOMP is a set of different phenomena allowing release (or leakage, see below) of apoptogenic factors such as cytochrome *c*, SMAC/diablo, AIF, through mitochondrial membrane pores. Cytochrome *c* received most attention for its ability to nucleate the apoptosome and to initiate the caspase cascade; its release occurs through at least two different mechanisms, the apoptosis-specific Bax-based pore, and the PTP channel, both of which can be modulated by Ca^2+^ in a very different way.

The relation between Ca^2+^ and cytochrome *c* release via Bax consists of a feed-forward amplification loop between ER and mitochondria: local high concentrations of Ca^2+^ (such as those created by the Ca^2+^ efflux from IP_3_ channels) favor the release of cytochrome *c* from mitochondria through Bax pores [62, 71] on the one side; on the other, cytosolic cytochrome *c* increases Ca^2+^ levels in the vicinity of IP_3_ channels on ER [38] by fixing them in the open configuration after a signaling stimulus, thus transforming a transient flux into a sustained one [38]. As a result of this interplay, small cytosolic cytochrome *c* leakage may promote secondary and massive releases (i.e., that required for apoptosome nucleation), via local Ca^2+^ messages [38]. This provides a rationale to previous reports indicating that small amounts of cytochrome *c* are released from mitochondria as a very early step of apoptosis, with the goal of expanding the signal [72]. Intriguingly, the Ca^2+^ sensitivity of Bax mitochondrial pores seems to be limited to the intrinsic pathway: when Bax is activated by the extrinsic pathway via t-Bid [73] (i.e., death receptor stimulation in type 2 cell), the Bax pores are insensitive to Ca^2+^ modulation [62]. This indicates that active Bax is different according to the route of activation (i.e., damage versus t-Bid), suggesting that multiple, alternative mechanisms for Bax activation may exist, possibly leading to different effects on the folding and functions of the protein [68, 70, 73].

PTP opening is an automatic response to excess [Ca^2+^]mt, which causes the interaction between the inner mitochondrial membrane complex adenine nucleotide translocator (ANT) and the outer mitochondrial membrane complex voltage-dependent anion channels (VDAC), leading to the formation of the membrane-spanning PTP pore. Cyclophilin D (Cyp-D) is a component of PTP resident in the mitochondrial matrix, which is activated by high [Ca^2+^]mt, favoring PTP opening by lowering the Ca^2+^ threshold required for ANT-VDAC interaction [74–76]. In instances of mitochondrial Ca^2+^ overload, inhibitors of Cyp-D activation, such as cyclosporins, contrast PTP opening and the eventual cell death, therefore exerting a net cell protective effect, which is often used in therapies to limit immune deficiencies or neurodegenerations [76, 77]. 

The mechanism of cytochrome *c* release via PTP opening, which was historically the first mechanism proposed, is still unclear from the molecular and functional point of view (Figure 1). In fact, there is a topological problem. In apoptosis cytochrome *c* is liberated from its natural position on the outer side of the internal mitochondrial membrane to the intermembrane space; thus, it requires pores in the outer membrane to be released, whereas pores that span the two membranes, such as PTP, would lead to the release of molecules residing in the mitochondrial matrix. To explain cytochrome *c* release via PTP, it may be hypothesized that PTP may cause mitochondrial membrane perturbations that allow cytochrome *c* (and other factors) to leak rather than be specifically released. In such instances, the gross alterations caused by PTP-mediated ionic redistribution will be necrogenic [78], even if cytosolic re-localization of cytochrome *c* may circumstantially activate caspases. Conceivably, the extent of PTP may determine the final outcome, and apoptosis or necrosis may follow according to the strength of PTP. Indeed, Cyp-D inhibitors are often reported to prevent cell death by necrosis. As an alternative mechanism of cytochrome *c* relase via PTP, it was suggested that a VDAC-only channel may form on the outer membrane, with the help, but without the physical participation, of ANT, thus connecting the cytosol not with the matrix, as in the canonical PTP, but with the intermembrane space, thus allowing cytochrome *c* release. This model is supported by experiments performed in liver mitochondria from mice knock out for ANT isoforms [79], where the release of cytochrome *c* following an apoptogenic stimulus still occurred, but the susceptibility to Ca^2+^ alterations in the mitochondrial matrix was reduced. In this instance ANT, a sensor of Ca^2+^ through its interaction with Cyp-D, plays the regulatory function to transduce Ca^2+^ alterations to VDAC, promoting its oligomerization and the formation of pores mediating release of cytochrome *c*.

Bax pores and PTP are different in molecular, mechanistic and functional term. However, they cooperate in some examples of apoptosis to achieve cytochrome *c* release. This implies physical interaction between Bax and PTP components [80], such as Cyp-D or ANT [81]. The two mechanisms of cytochrome *c* release also coparticipates in the same induction pathway in a different temporal relationship, that is, a mild stress-induced PTP opening first causes a small cytochrome *c* leakage, which stimulates via Ca^2+^ modulation (see above) a second intense Bax-mediated release sufficient for caspase activation. The co-operation between the two pores (and the two pathways) also provides a mechanistic explanation to the established but still unexplained finding that Bcl-2, though not modulating PTP directly [74], all the same provides protection to cells against necrosis [82].

### 3.4. Control of Ca^2+^ by the Bcl-2 Family

The pro-apoptotic protein Bax exerts its functions by inserting into membranes and forming pores. Very well described is the anchoring to mitochondrial membrane, where Bax forms, perhaps with adjuvant proteins, pores of a size large enough to allow passage of diffusible pro-apoptotic proteins such as cytochrome *c* or SMAC [83–85]; the anti-apoptotic role of the cognate Bcl-2, which integrates into mitochondrial membranes also in healthy cells, is believed to be the prevention of Bax pore forming, perhaps due to the extra protein domain (BH4) shared by all anti-apoptotic members of the family. It is now emerging that Bax and Bcl-2 play a similar role also in the ER membranes, where they would prevent or favor, respectively, Ca^2+^ leakage to the cytosol.

Bcl-2 is found within ER membranes of healthy cells, where it prevents Ca^2+^ leaking from ER [63, 86]; as the mechanism involved, it was proposed that Bcl-2 may work as a pump additional to the SERCAs [56], and/or to prevent IP_3_ channels opening [87]. 

In apoptosis, Bax translocates not only to mitochondria, but also to ER membranes [63, 88], where it favors Ca^2+^ release from the ER lumen [62, 63], possibly after oligomerization [71]; similar evidence was shown for the cognate pro-apoptotic Bak [63, 88]. The mechanism through which Bax (and Bak) favors ER Ca^2+^ release is currently debated. Recent cell-free studies have shown that Bax forms small pores, compatible with multi-ion passage, on membranes [89], thus possibly directly allowing Ca^2+^ leakage. Other studies indicate an indirect role, that is, favoring IP_3_ channels opening [90, 91]. Ca^2+^ release from ER in turn favors the recruitment of more Bax molecules from the cytosol to ER membranes [88], thus amplifying the Ca^2+^-dependent apoptotic signal [71]. Thus, the pro-apoptotic functions of Bax and Bak are not limited to mitochondria, but consist of a dual concerted role played at an earlier pre-commitment step at the level of ER membranes, promoting Ca^2+^ movements; and at a later step in mitochondria to promote MOMP and the release of the apoptotic factors. It remains to be clarified if the mechanism of Bax translocation to ER in pre-apoptosis occurs with the same mechanism as mitochondrial translocation, that is, if there is a role for t-Bid or other BH3-only proteins of the Bcl-2 family; moreover, it is still unclear if Bax domains involved in promoting ER Ca^2+^ leakage coincide with those required for releasing the apoptotic factors from mitochondria.

### 3.5. Calpain and Apoptosis

A role for the cysteine proteases calpains, which are activated by Ca^2+^ increase, was investigated since the earlier studies of apoptotic signaling, considering that (a) cytosolic Ca^2+^ overload was then considered as the main mediator of apoptosis [92], and (b) apoptosis might be conceivably executed by coordinate protein dismantling (which was later demonstrated for caspases) [58, 93]. The focus was placed on the known calpain target fodrin [94], the protein bridging plasma membrane with the cortical actin cytoskeleton; it was hypothesized that fodrin degradation might destabilize the cytoskeleton-membrane asset and promote plasma membrane blebbing [95], one of the earliest apoptotic features described [96]; however, such evidences have not been confirmed; actually, calpain activation seems rather inhibiting plasma membrane blebbing (De Nicola et. al, in preparation). 

Nowadays, many pieces of evidence show that calpains are required for apoptosis in some systems [97–100], being dispensable (or not involved) in others [101]; when calpains are involved, they act at a very early step, upstream of caspases [100, 102], thus participating to the commitment phase of signaling rather than to the execution. The notion that the form of calpain involved in apoptosis is m-calpain [103], the one also involved in cell stress [20] and that requires high (mM) Ca^2+^ levels (as opposed to *μ*-calpain, involved in cell signaling, and requiring lower, *μ*M doses) was very important because it allowed linking environmental alterations to apoptosis via Ca^2+^ overload. The molecular role for calpain in promoting apoptosis is still under investigation. Perhaps the most clear-cut hint is the calpain-mediated proteolytic Bax activation [70, 104], one of the few mechanisms so far proposed for direct Bax activation by cell damage [104–106]. Two mitochondrial calpains co-operate in the release of a truncated active form of AIF (tAIF) thus promoting apoptosis: a matrix m-calpain cleaves AIF [107]; and a transmembrane *μ*-calpain cleaves VDAC, promoting the formation of Bax-VDAC pores on the outer mitochondrial membrane and the release of tAIF [108]. 

A complex interplay between calpain and caspases occurs in apoptosis. Calpain have been proposed to proteolytically activate some caspases [100, 109–112]; paradoxically, caspases may also be degraded by calpains [113], which in such instances would act to prevent, rather than promote, apoptosis; the factors influencing this discrepant behavior have not been clarified.

## 4. Ca^2+^ and the Stress Response

### 4.1. Generalities

Stress consists of any physico-chemical alteration of cell environment that interferes with cell functioning, potentially or actually producing damage. Stress elicits active cell responses that, according to cell type, and to type and extent of damage, aim at cell survival (cell-protective and/or cell-reparative stress response, such as the heat shock response) or cell death (apoptosis). 

Stress responses are specific for a given type of alteration/damage: heat, oxidation, hypoxia, starvation, all trigger the synthesis or activation of molecular determinants adequate to cope with the specific type of damage; for example, heat shock will induce synthesis of molecular chaperones to cope with exposure of hydrophobic residues of proteins [114]; oxidative stress will induce the synthesis/activation of anti-oxidant enzymes or molecules [115]; hypoxia promotes anaerobic metabolism [116]; starvation promotes the disassembly of whole cell areas that are digested by autophagy [117], in order to recycle the building blocks for housekeeping purposes. This specificity of response limits the cross-resistance between different stress, even though a partial overlapping exist.

A brief/mild insult is often sufficient to trigger protective responses but not to produce damage. This protects the cells from a second, more severe insult of the same type, thus producing transient tolerance to further stress, as occurring, for example, during thermotolerance [118]. Treatments with important clinical relevance, such as ischemia preconditioning, that is a short anoxic treatment that is protective towards a more severe ischemia, and whose mechanisms are still to be elucidated at the molecular level, seem to depend on Ca^2+^ signaling [119]. Ca^2+^ participates as second messenger to such defensive, reparative, or survival pathways, propagating the cell-protective signals. 

High [Ca^2+^]c is involved in the stimulation of the autophagic response through the activation of calcium/calmodulin-dependent kinase-b that inhibits mTOR [120], the main negative regulator of authophagic processes in mammals, with the goal of eliminating cellular areas that may be damaged by Ca^2+^ overload.

### 4.2. Ca^2+^ in Cell Survival

In addition to these specific stress responses, cells are capable to build up survival pathways that render them less prone to apoptosis, thus promoting cell survival whatever the type of damage; this especially occurs in cells that reside in highly stressing environments, such as inflammatory or immune cells while exerting their functions [121], or transformed cells undergoing tumor progression [122], process in which cells carrying apoptosis-resistant mutations are favored by natural selection.

Ca^2+^ is involved in pro-survival or anti-apoptotic pathways, such as the activation of protein kinase C, whose many isoforms play pivot roles in coordinating survival cell responses [123].

Capacitative Ca^2+^ entry (CCE) is Ca^2+^ influx from the extracellular environment through specific and tightly controlled plasma membrane channels [4, 5]. CCE only transiently crosses the cytosol, its aims being rather the replenishment of ER, after it was partially emptied by signaling events such as cyclic ADP-ribose- [4] or IP_3_-mediated opening of ER Ca^2+^ channels [5]. CCE poorly alters cytosolic homeostasis, but prevents ER vesiculation due to Ca^2+^ emptying, thus being a net cell-protective event. 

**Figure 2:**
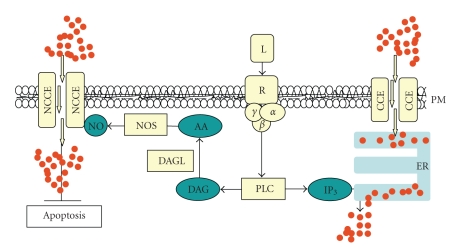
*Capacitative and noncapacitative Ca^2+^ entry.* Ligand (L) stimulation of G-protein (*α* *β* *γ*)-coupled receptor (R) activates phospholipase C (PLC) to produce diacylglycerol (DAG) and inositol-3-phosphate (IP_3_). IP_3_ (right side) causes ER Ca^2+^ emptying, eliciting a capacitative Ca^2+^ entry (CCE) through plasma membrane (PM), aimed at refilling ER of Ca^2+^ restoring ER homeostasis. DAG (left side) is processed to arachidonic acid (AA) by DAG lipase (DAGL), stimulating NOS to produce NO, which activates Ca^2+^ entry through PM by a noncapacitative Ca^2+^ entry (NCCE), priming specific signaling including anti-apoptotic pathways. CCE and NCCE differ in protein composition [124]. Red dots symbolize Ca^2+^.

Recently, another mechanism of Ca^2+^ influx is being considered, namely the noncapacitative Ca^2+^ influx (NCCE) [124], a non-store-operated mechanism that allows Ca^2+^ entry through plasma membrane channels that are different from those of CCE from the molecular and regulative point of view. As depicted in Figure 2, NCCE, as CCE, occurs as a response to receptor stimulation implying G-protein and phospholipase C (PLC) but, unlike CCE, it does not respond to IP_3_-induced ER emptying; instead, it results from the processing of diacylglycerol (the other product of inositol-bis-phosphate cleavage by PLC), which promotes a signal transduction chain culminating with NCCE assembly. Interestingly, NCCE occurs also in non-excitable cells. Even though mechanisms and functions are still poorly characterized, it is clear that Ca^2+^ entry via NCCE has a signaling function, possibly implying the control of survival pathways. Interestingly, it requires production of NO, a molecule that is involved in many survival pathways [125], including a strict interrelationship with protein kinase C [124]. Agents promoting cell survival such as magnetic fields reduce stress-induced apoptosis by increasing Ca^2+^ influx [126, 127], involving NCCE rather than CCE (Cerella and Ghibelli, in preparation).

### 4.3. Stand-By Mechanisms

**Figure 3:**
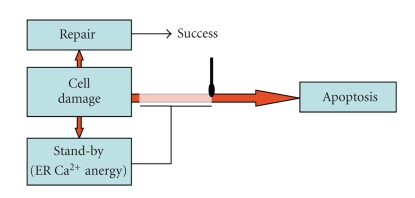
*Temporary ER Ca^2+^ anergy avoid loss of repairable cells.* Cell damage elicits repair and apoptosis as well as standby periods (red arrows). ER Ca^2+^ anergy is temporarily achieved via ADP-ribosylation of GAPDH, glycolysis block and starvation of Ca^2+^-ATPases, and hampers apoptotic signal transduction at the ER signaling stage. After resumption of glycolysis and ER Ca^2+^ activity, the apoptotic signal is allowed to proceed, unless successful repair has occurred in the meantime.

The decision between cell repair or demise of damaged cells is a choice between the risk of mal-repair, leading to stabilize mutations and potentially preserve precancerous cells, versus loss of viable cells performing useful functions, which must be expensively replaced. Even though the former risk is definitely worse than the latter, mechanisms aiming at avoiding unnecessary loss of precious cells have evolved. To this purpose, it is important that damaged cells do not initiate apoptosis before attempting to repair the damage: this is actively achieved by damaged cells via the set-up of reversible standby scenarios, during which apoptotic signaling is transiently kept at bay. One of such standby mechanisms implies that potentially apoptogenic stress conditions such as H_2_O_2_ treatment cause the transient inhibition of glycolysis mediated by the reversible ADP-ribosylation of glyceraldehyde-3-phosphate-dehydrogenase (GAPDH) [128]; this inactivates ER Ca^2+^-ATPases, which are fed by glycolytic ATP [129], thus decreasing ER Ca^2+^ while increasing [Ca^2+^]c and impairing Ca^2+^-mediated cell signaling. Many pieces of evidence from the literature show that cells with partial Ca^2+^-depleted ER cannot initiate apoptosis [130–132], and indeed, during the standby period of glycolysis block, apoptosis cannot initiate [128]. Starting at around 90 minutes after recovering from H_2_O_2_ stress, glycolysis resumes [128], ER Ca^2+^ increases, and [Ca^2+^]c is reduced [64]; only then H_2_O_2_-induced apoptosis begins [128]; (Cerella et al., in preparation). Similar findings were reported also for other, oxidation-unrelated apoptogenic agents, strongly supporting the scenario according to which (a) Ca^2+^ signaling is required for stress-induced apoptosis [64], and (b) ER is the initiator of the apoptotic signaling, since the standby phase seems to prevent ER from amplifying apoptotic signal and mitochondrial recruitment. A model representing the relationship between apoptogenic, repair and standby signals in damaged cells is shown in Figure 3.

## 5. Conclusions

**Figure 4:**
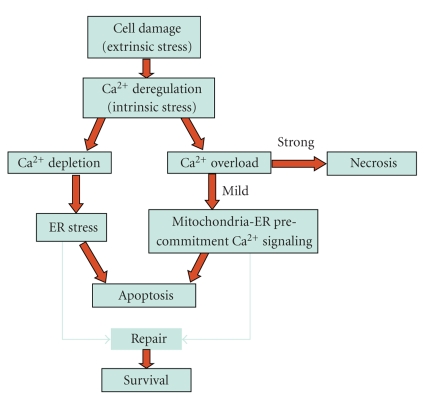
Ca^2+^ signaling in survival and apoptosis versus deregulation of Ca^2+^ homeostasis as necrogenic event.

The relationship between Ca^2+^ and cell death has a long and complex story. It was a reasonably simple task when the goal was describing how strongly deregulated intracellular Ca^2+^ may cause the passive cell death by necrosis. The scenario became very much complex when the increasing information of the mechanisms of Ca^2+^-mediated cell signaling in general, and apoptotic signaling in particular, begun to merge. Figure 4 depicts the different roles that Ca^2+^ alterations, as an intrinsic stressor, play in the survival or death of damaged cells, aiming at separating Ca^2+^ deregulation from pro-apoptotic Ca^2+^ signaling. Perhaps the hottest topic to-date in this field is the role that ER, and the Ca^2+^ messages it exchanges with mitochondria, plays in the amplification of the apoptotic signal, ending up with the promotion of MOMP and the trigger of the commitment phase of the intrinsic apoptotic signaling. The amplification loops created by the concerted action of ER, Bax, IP_3_ channels and cytochrome *c* with Ca^2+^ signals, spanning ER and mitochondria via cytosol, as depicted in Figure 1, are beginning to define a novel precommitment phase of apoptosis. This is a very important issue because, unlike the extrinsic apoptotic pathway, which has been very well characterized at the molecular level since many years, the molecular events of the intrinsic pathway upstream of MOMP are poorly understood. From the functional point of view, a precommitment phase might have the role of selecting, among the pro-apoptotic signals deriving from cell damage, the ones that have to be finalized in cell death, thus avoiding unnecessary cell loss. Unlike receptor-induced apoptosis, before commitment to damage-induced apoptosis cells must check the extent of the damage, and the possibility to repair it, before engaging the apoptotic signaling and commit suicide. To this purpose, different signals reporting the nature and the extent of the damage must merge into a mainstream signal that actually allows the onset of apoptosis, which in molecular terms coincides with MOMP. Many evidences allow proposing the fascinating scenario according to which ER plays as a pivot that receives the damage signals and select those that actually deserve ending up in apoptosis.

The acknowledgement of a Ca^2+^-dependent pre-commitment apoptotic phase would place Ca^2+^-related events among the earliest of apoptosis, which would make the closing of a circle that begun almost 20 years ago, when Ca^2+^ as an intrinsic stressor was considered as “the” mediator of apoptosis.

## Abbreviations

AA: Arachidonic acid
AIF: Apoptosis inducing factor
ANT: Adenine nucleotide translocator
CCE: Capacitative calcium entry
ER: Endoplasmic reticulum
GAPDH: Glycerhaldehyde-3-phospahate dehydrogenase
IP_3_: Inositol-3-phosphate
MOMP: Mitochondrial outer membrane permeabilization
NCCE: Non-capacitative calcium entry
PLC: Phospholipase C
PTP: Permeability transition pore
SERCA: Sarcoplasmic/endoplasmic reticulum calcium ATPases
THG: Thapsigargin
VDAC: Voltage-dependent anion channels.
